# CHARACTERISTICS OF MIDLIFE WOMEN FAMILY CAREGIVERS ASSOCIATED WITH THEIR NEGATIVE ATTITUDES TO DEMENTIA AND CARE

**DOI:** 10.1093/geroni/igae098.1494

**Published:** 2024-12-31

**Authors:** Dongmi Kim, Eun-Ok Im, Wonshik Chee, You Lee Yang

**Affiliations:** The University of Texas Austin, Austin, Texas, United States; The University of Texas Austin, Austin, Texas, United States; The University of Texas Austin, Austin, Texas, United States; Eulji University, Eulji University, Kyonggi-do, Republic of Korea

## Abstract

Racial/ethnic minority family caregivers, especially midlife women who are family caregivers of persons living with Alzheimer’s Disease (MWPLAD), tend to suffer quietly without necessary support and care partially due to their cultural attitudes related to Alzheimer’s Disease (AD) and dementia care (e.g., filial piety, fatalism). Yet, little is still known about the women’s characteristics that are associated with their negative attitudes toward AD and dementia care. This information could provide important directions for future intervention development for this specific population of family caregivers. The purpose of this secondary analysis study was to determine the characteristics of MWPLAD that were significantly associated with their negative attitudes toward AD and family Caregiving. This analysis included the data from 172 MWPLAD who were recruited through online and offline communities/groups for midlife women. The instruments included: the Attitude toward AD and Related Dementias Scale (DAS), the Questions on Attitudes toward AD Caregiving, the Social Readjustment Rating Scale, the EQ-5D-5L and the Midlife Women’s Symptom Index. The data analysis was conducted with descriptive and inferential statistics including Independent t-tests, Mann Whitney U tests, one-way ANOVA, and multiple regression analyses. The total DAS scores were significantly associated with employment, race/ethnicity (being Hispanic), and instrumental activities for daily living (p <.05). Those who were unemployed, Hispanic, and with low instrumental activities for daily living tended to have negative attitudes toward Alzheimer’s Disease and dementia care. More interventions need to be developed for racial/ethnic minority MWPLAD while considering these characteristics.

